# Association between blood metals mixtures concentrations and cognitive performance, and effect modification by diet in older US adults

**DOI:** 10.1097/EE9.0000000000000192

**Published:** 2022-01-25

**Authors:** Nasser Laouali, Tarik Benmarhnia, Bruce P. Lanphear, Jennifer Weuve, Michael Mascari, Marie-Christine Boutron-Ruault, Youssef Oulhote

**Affiliations:** a Department of Biostatistics and Epidemiology, School of Public Health and Health Sciences, University of Massachusetts at Amherst, Amherst, Massachusetts; b Department of Family Medicine and Public Health & Scripps Institution of Oceanography, University of California, San Diego, California; c CESP UMR1018, Université Paris-Saclay, UVSQ, Inserm, Gustave Roussy, Villejuif, Paris, France; d Child and Family Research Institute, BC Children’s Hospital, Vancouver, British Columbia, Canada; e Faculty of Health Sciences, Simon Fraser University, Burnaby, British Columbia, Canada; f Department of Epidemiology, Boston University School of Public Health, Boston, Massachusetts

**Keywords:** Adapted Dietary Inflammatory Index, Cadmium, Epidemiology, Healthy Eating Index, Lead, Manganese, National Health and Nutrition Examination Survey

## Abstract

**Background:**

Chronic exposure to heavy metals has been associated with adverse neurological outcomes in older adults. Inflammatory processes are suspected as an underlying pathway by which metals exert their neurotoxicity. In parallel, a diet rich in antioxidant and anti-inflammatory components may protect against chronic inflammation.

**Objectives:**

We examined the associations of blood concentrations of lead, cadmium, and manganese as a mixture with cognitive performance in older US adults and potential modification of these associations by diet as measured by the Healthy Eating Index 2015 (HEI-2015) and the Adapted Dietary Inflammatory Index (ADII).

**Methods:**

We used data on 1,777 adults ≥60 years old from the US National Health and Nutrition Examination Survey (NHANES; 2011–2014). We derived the ADII and the HEI-2015 from two nonconsecutive 24-hour diet recalls. Cognitive performance was measured by the Consortium to Establish a Registry for Alzheimer’s Disease (CERAD) Word Learning subtest, the animal fluency test, and the Digit Symbol Substitution Test (DSST). We also constructed a composite z-score reflecting overall cognitive performance. We used quantile g-computation to evaluate the joint associations of a mixture of metals with cognitive performance test scores. We also evaluated effect modification by sex and diet quality indices using Cochran Q tests.

**Results:**

The median (interquartile range) of blood metals were 0.38 μg/L (0.35), 14.70 μg/L (11.70), and 8.74 μg/L (4.06) for cadmium, lead, and manganese, respectively. Increasing blood concentrations of all metals by one quartile was associated with a decrease in overall cognitive performance (–0.04; 95% confidence interval [CI] = –0.09, 0.02), CERAD (–0.04; 95% CI = –0.12, 0.03), animal fluency (–0.02; 95% CI, –0.11, 0.06), and DSST (–0.05; 95% CI = –0.11, 0.02) test scores. These associations were more pronounced in adults with high pro-inflammatory or low-diet quality and null or positive though imprecise associations in participants with a high anti-inflammatory. These associations also varied by sex with inverse associations in men and positive associations in women.

**Conclusions:**

Our findings suggest that adherence to an antioxidant and anti-inflammatory diet may prevent blood metals adverse cognitive effects among older adults. If confirmed, strategies based on diet could provide a potential complementary and efficient approach to counteract effects of environmental pollutants.

**What this study adds**

Chronic exposure to heavy metals has been associated with adverse neurological outcomes in older adults and inflammatory processes are suspected as an underlying pathway. In parallel, a diet rich in antioxidant and anti-inflammatory components may protect against chronic inflammation. However, to the best of our knowledge, no study has evaluated the potential modification of the associations between metals and adverse neurological outcomes by dietary scores. In this cross-sectional study, we found that an antioxidant and anti-inflammatory diet may mitigate adverse cognitive effects of metals among older adults. This result provides a potential complementary and efficient approach to counteract effects of environmental pollutants.

## Introduction

Aging is a naturally occurring physiological process that is characterized by both physical and cognitive decline. Cognitive decline is a process characterized by a gradual deterioration from mid-adulthood onwards in cognitive functions including processing speed, reasoning, and memory and executive functions.^1^ In addition to advanced age, other factors may contribute to declines in cognitive performance. Gurland et al^2^ estimated that 25% of inter-individual variability is due to genetic factors, leaving a large unexplained proportion potentially due to modifiable risk factors, including gene-environment interactions.^3^ With the increases in life expectancy worldwide, there is a strong interest in identifying modifiable factors that accelerate cognitive decline. As such, recent research has focused on understanding environmental factors, including exposure to neurotoxic metals^4–6^ and lifestyle factor, such as dietary quality.^7^

Heavy metals, such as lead (Pb), manganese (Mn), and cadmium (Cd), are naturally present in our environment. Overall, cross-sectional and longitudinal studies suggest that cumulative or long-term exposure to these metals, measured where they are stored, was associated with cognitive decline and impaired cognitive function in older adults.^8–11^ However, the evidence is less consistent when it comes to measures of current exposure, such as measurements in blood.^12–14^ Although the underlying mechanisms are not completely understood, metals may exert their neurotoxicity via. inflammatory processes in the brain.^15^ Several heavy metals are involved in the oxidant/antioxidant mechanisms through the production of free radicals, thus leading to oxidative stress,^16,17^ impairment of endothelial cell function, and the initiation of inflammatory processes.^18^ On the other hand, inflammatory processes and oxidative stress can also be promoted and prevented by nutritional behaviors and food constituents. Some foods and nutrients (e.g., fruits and vegetables, omega-3 polyunsaturated fatty acid, folic acid) may reduce the inflammation and oxidative stress through their radical-scavenging capacity and inhibition of the production of pro-inflammatory cytokines.^19,20^ More interestingly, a recent study reported that dietary antioxidant and anti-inflammatory intake modifies the effect of cadmium exposure on markers of systemic inflammation and oxidative stress.^21^

Although heavy metals and dietary intake are both involved in the regulation of inflammation and cognitive performance, their contribution to cognitive performance in older adults is poorly understood. Recent findings suggest a potential protective effect of folic acid, an antioxidant component of the diet; against effects of phthalate metabolites,^22^ pesticides^23^ and air pollutants^24^on autistic traits^24^ and intellectual abilities in children,^25^ or on the risk of preterm birth.^26^ However, to our knowledge, no study evaluated similar patterns for other environmental contaminants on cognitive performance among the elderly. In this investigation, we hypothesized that a high-diet quality or adherence to an anti-inflammatory dietary pattern may attenuate inflammation and subsequently reduce the metals-induced neurotoxicity. Therefore, we sought to examine the associations of a mixture of metals with the cognitive performance in US older adults and the potential effect modification by overall diet quality as measured by the Healthy Eating Index 2015 (HEI-2015) and the Adapted Dietary Inflammatory Index (ADII).

## Methods

### Design and participants

We used data from the US National Health and Nutrition Examination Survey (NHANES). The NHANES is an ongoing survey conducted by the Centers for Disease Control and Prevention (CDC) that uses a representative sample of noninstitutionalized civilians in the United States; selected by a complex, multistage, stratified, clustered probability design. Trained staff collected information on participants by interviews and physical examinations. The interview includes background information such as sociodemographic, dietary, and health-related questions. The examination component consists of medical and physiological measurements, and laboratory tests. The National Center for Health Statistics Ethics Review Board approved all NHANES protocols, and all survey participants completed a consent form. The detailed protocol on NHANES methodology and data collection is available on https://www.cdc.gov/nchs/nhanes/index.htm. For this study, we included participants with data on blood metals concentrations (n = 2,068). We excluded participants with missing data on diet (n = 291). The final study population included 1,777 adults ≥60 years old with data on dietary intake who participated in the cognitive function test of the 2011–2012 and 2013–2014 cycles.

### Measurements of trace metals in blood

The methodological details on the laboratory analyses are described in detail on the NHANES website. Briefly, whole blood specimens were frozen (–30 °C), stored, and shipped for analysis to the Division of Laboratory Sciences, National Center for Environmental Health of the CDC. Cadmium (µg/L), lead (µg/dL), and manganese (µg/L) concentrations were measured in whole blood using inductively coupled plasma mass spectrometry. This multielement analytical technique is based on quadrupole inductively coupled plasma mass spectrometry technology. Coupling radio frequency power into a flowing argon stream seeded with electrons creates the plasma. Predominate species in the plasma are positive argon ions and electrons. Diluted whole blood samples are converted into an aerosol using a nebulizer inserted within a spray chamber. A portion of the aerosol is transported through the spray chamber and then through the central channel of the plasma, where it experiences temperatures of 6,000–8,000 °K. This thermal energy atomizes and ionizes the sample. The ions, along with the argon, enter the mass spectrometer through an interface that separates the inductively coupled plasma, operating at atmospheric pressure (approximately 760 torr), from the mass spectrometer, operating at approximately 10^–5^ torr. The lower limits of detection (LOD) in 2011–2012 and 2013–2014 cycles were 0.16 μg/L and 0.10 μg/L for cadmium, 0.25 μg/dL and 0.07 μg/dL for lead, and 1.06 μg/L and 0.99 μg/L for manganese. Measures below LOD were set by NHANES as LOD/√2. In 2011–2012 and 2013–2014 cycles, LOD constituted 31% and 29% for cadmium, 1% and 0% for lead, and 0% in both cycles for manganese.

### Evaluation of cognitive performance

Cognitive performance was measured by the Consortium to Establish a Registry for Alzheimer’s Disease (CERAD) Word List Learning and Recall Test, the Animal Fluency test, and the Digit Symbol Substitution Test (DSST). The CERAD Word List Learning and Recall Tests were designed to assess immediate and delayed learning ability for new verbal information.^27^ For the learning trials, participants were instructed to read aloud 10 unrelated words, one at a time, as they were presented. Immediately following the presentation of the words, participants recalled as many words as possible. In each of the three learning trials, the order of the 10 words is changed. The delayed word recall occurred after the other two cognitive exercises (Animal Fluency and DSST) were completed (approximately 8–10 minutes from the start of the word learning trials). The maximum score possible on each trial is 10. The Animal Fluency test examines categorical verbal fluency, a component of executive function in which participants were asked to name as many animals as possible in 1 minute.^28^ The DSST, a performance module from the Wechsler Adult Intelligence Scale, was used to assess processing speed, sustained attention and working memory.^29^ The exercise was conducted using a paper form that has a key at the top containing nine numbers paired with symbols. Participants have 2 minutes to copy the corresponding symbols in the 133 boxes that adjoin the numbers. The score is the total number of correct matches. Higher scores represent better cognitive function for all tests. In addition, we calculated a composite z-score reflecting the overall cognitive performance as suggested by Li et al.^4^ This composite cognitive z-score was the average of the standardized scores of the CERAD Word List Learning and Recall Test, the DSST, and the Animal Fluency test. All cognitive test scores were standardized to keep the same scale as the composite score. Higher scores represent better cognitive function for all tests.

### Dietary data and diet quality indices

Dietary intake was collected from the two NHANES cycles using two 24-hour dietary recalls. The first 24-hour recall interview was conducted at a Mobile Examination Center using a standard set of measuring guides to assist in estimating portion sizes. The second dietary recall, which was collected by telephone, was scheduled 3 to 10 days later. We derived the ADII and the HEI-2015 from the mean daily intakes of foods/beverages, energy, and nutrients of these two nonconsecutive 24-hour dietary recalls.

The Adapted Dietary Inflammatory Index (ADII) was constructed to measure the inflammatory potential of the diet.^30^ Briefly, the ADII index proposed by Van Woudenbergh et al^31^ was used in combination with the updated dietary components inflammatory weights designed by Shivappa et al^32^ instead of the weights proposed by Cavicchia et al.^33^ These updated inflammatory weights, based on 3 additional years of published data (2008–2010, inclusive), resulted in a doubling of the total number of articles scored. The ADII was based on a nutritional rationale: first, the inflammatory weights of dietary components are multiplied by the standardized energy-adjusted intake,^34^ which acts to reduce between-person variation; second, the intakes of all components are standardized; third, the ADII calculation did not include alcoholic beverages such as beer, wine, and liquor, total fat, and energy, to avoid overestimation of the inflammatory effects of ethanol, fat, and energy. A total of 24 of the 35 possible dietary components were used for the ADII calculation (carbohydrates, proteins, alcohol, fibers, cholesterol, saturated fatty acids, monounsaturated fatty acids, omega 3, omega 6, niacin, thiamin, riboflavin, vitamin B6, vitamin B12, iron, magnesium, zinc, vitamin A, vitamin C, vitamin D, vitamin E, folic acid, beta carotene, and caffeine). A positive ADII score indicates an anti-inflammatory diet and negative values correspond to a pro-inflammatory diet.

The Healthy Eating Index 2015 (HEI-2015) was developed to measure overall diet quality.^35,36^ Briefly, HEI-2015 is a composite measure of conformance to the 2015–2020 Dietary Guidelines for Americans and has been well validated in the US population.^37^ The adequacy components (maximum score) were total fruits (5), whole fruits (5), total vegetables (5), greens and beans (5), whole grains (10), dairy (10), total protein foods (5), seafood and plant proteins (5), and fatty acids (ratio of the sum of polyunsaturated and monounsaturated fatty acids to saturated fatty acids, 10). The moderation components were refined grains (10), sodium (10), added sugars (10), and saturated fats (10). HEI-2015 is a 100-point scale, with a higher score indicating a better quality of overall diet.

### Covariates

We included participants’ age (continuous; years), race/ethnicity (non-Hispanic White, non-Hispanic Black, Mexican American, other Hispanic, or other/multiracial), sex (male/female), language of the sampled person interview instrument (English, Spanish), educational level (less than high school, high school, higher than high school, and in number of years), smoking status (current, former and never), and family income to poverty ratio (in quartiles) in the analyses. There were no missing values for all variables except for poverty to income ratio (8%); thus, an “unknown” category was created to keep the same number of participants in all models. Models were adjusted for age, sex, marital status, race/ethnicity, family income to poverty ratio, language of the sample person interview instrument, educational level, and diet quality evaluated by the healthy eating index. All these covariates were included in the models as potential confounders and were selected a priori based on previous literature.

### Statistical analyses

Means and SDs, and medians and interquartile ranges (IQRs) were calculated for cognitive performance tests scores and blood metals concentrations and also presented by population characteristics. All blood metals concentrations were log_10_-transformed to reduce the influence of outliers.

We used quantile g-computation to evaluate the joint mixture association of the three blood metals concentrations with the cognitive performance tests scores. This method is a parametric, generalized linear model-based implementation of g-computation. Quantile g-computation is adapted from weighted quantile sum regression, but unlike weighted quantile sum, this method is not constrained to a single direction of effect, does not require splitting of the dataset, and is more robust against unmeasured confounders.^38,39^ In addition, quantile g-computation yields a simple and computationally efficient approach to estimating associations between a mixture of exposures and a health outcome of interest, when we may not be able to rule out confounding or we may be uncertain about the effect direction of some exposures in the mixture.^39^ The quantile g-computation allows us to estimate the expected difference in the cognitive performance test scores when simultaneously increasing all blood metals concentrations by one quantile, conditional on covariates. We used quartiles and derived 95% confidence intervals (CIs) of the estimates using the bootstrap method (N = 200). We assigned each metal a weight that was then used in a multivariable linear regression model for each cognitive test score, conditional on covariates listed above. The weights can either be positive or negative and reflect the individual contribution of each metal to the overall mixture effect. We also present metals independent effects on cognitive test scores using traditional multivariable regressions in Supplementary Table 1 (http://links.lww.com/EE/A173).

We also investigated sex-specific estimates in stratified analyses because prior studies reported sex-specific associations between metals concentrations and cognitive function.^40,41^ We also examined dietary modification of the association between metals mixtures and cognitive test scores. To examine differences between estimates from different sex- and dietary-specific strata, we used the method developed by Payton et al.^42^ This method allows examination of overlap between confidence intervals or standard error intervals to test hypotheses about the difference between estimates of dietary scores strata. We estimated 4-year survey weights for each participant by dividing the 2-year weights by 2 (the number of 2-year cycles NHANES 2011–2012 and 2013–2014) and used them in all analyses to account for the unequal probabilities of inclusion and response rates.

### Sensitivity analyses

We ran three sensitivity analyses. First, we performed unweighted analyses that did not account for the survey design because the weighted method is inefficient analysis due to the large variability in assigned weights.^43^ The unweighted analysis yields correct estimates when models are adjusted for the auxiliary variables used to define the weights (i.e., age, sex, and ethnicity).^43^ Second, we tested effect modification using another alternative definition of the dietary inflammatory index proposed by Shivappa et al.^32^ This score is based on the same inflammatory weights and foods components as in the ADII; however, the foods components were not energy-adjusted to reduce the between-person variation. Finally, the main analysis was restricted to never smokers.

## Results

### Descriptive statistics

The mean age of participants at screening was 69.5 years (SD = 6.7 years), with slightly fewer men (49%) than women (51%). The mean scores of CERAD, Animal Fluency, DSST, and the overall cognitive performance were 46.4 (SD = 17.1), 16.7 (SD = 5.4), 24.7 (SD = 6.4), and 87.8 (SD = 24.3), respectively. In general, women, non-Hispanic White, highly educated, younger (<69 years), and participants who were in the 4th quartile groups of HEI-2015 scores and poverty to income ratio, had higher scores of overall and specific cognitive performance tests. Additionally, participants in the 4th quartile group of ADII score—which corresponds to a higher anti-inflammatory diet—had higher test scores compared to participants in the 1st quartile (Table 1).

**Table 1.** Means of the overall cognitive function and the specific cognitive performance tests according to characteristics of study participants, NHANES 2011–2014

		Overall cognitive function	DSST test	CERAD test	Animal fluency test
Characteristics of participants	n (%)	Means (SD)	Means (SD)	Means (SD)	Means (SD)
Gender					
Male	869 (49)	83.7 (23.1)	23.3 (6.1)	43.6 (16.2)	16.8 (5.5)
Female	908 (51)	91.7 (24.7)	26.0 (6.4)	49.1 (17.5)	16.6 (5.3)
Age at screening (years)					
60–69	957 (54)	93.5 (24.4)	26.0 (6.0)	49.8 (17.4)	17.6 (5.6)
70–79	543 (31)	84.6 (22.8)	24.0 (6.4)	44.3 (16.2)	16.3 (5.2)
≥80	277 (15)	74.4 (20.0)	21.1 (6.3)	38.7 (14.1)	14.6 (4.4)
Education level					
Less than 9th grade	199 (11)	62.0 (16.0)	20.6 (5.9)	27.1 (11.0)	14.3 (4.3)
9–11th grade	232 (13)	75.0 (20.0)	23.1 (6.1)	37.3 (13.9)	14.7 (4.9)
High-school graduate	431 (24)	84.8 (21.5)	24.4 (6.1)	44.7 (15.0)	15.7 (4.9)
Some college or AA degree	498 (28)	95.4 (21.3)	26.0 (6.1)	51.8 (14.9)	17.6 (5.3)
College graduate or above	417 (24)	101.3 (22.1)	26.3 (6.4)	56.0 (15.1)	19.1 (5.7)
Poverty to income ratio					
Quartile 1	408 (23)	76.2 (22.6)	23.2 (6.4)	37.7 (16.0)	15.3 (5.2)
Quartile 2	410 (23)	82.8 (21.7)	24.0 (6.2)	42.7 (15.2)	16.1 (4.9)
Quartile 3	405 (23)	92.6 (21.5)	25.5 (6.3)	50.1 (14.6)	17.1 (5.3)
Quartile 4	413 (23)	101.2 (23.1)	26.2 (6.3)	56.3 (15.8)	18.8 (5.6)
Missing	141 (8)	82.7 (24.8)	24.1 (6.3)	42.9 (18.1)	15.6 (4.9)
Race/Hispanic origin					
Mexican American	137 (8)	79.7 (22.7)	23.5 (5.8)	40.1 (16.9)	16.1 (4.6)
Other Hispanic	181 (10)	75.3 (22.8)	22.9 (6.1)	36.8 (16.7)	15.7 (4.9)
Non-Hispanic White	897 (50)	94.0 (24.0)	25.0 (6.6)	51.0 (16.1)	18.0 (5.6)
Non-Hispanic Black	417 (24)	80.6 (22.8)	24.7 (6.3)	40.7 (15.9)	15.2 (5.1)
Other race—including multiracial	145 (8)	93.6 (19.6)	26.1 (5.9)	52.3 (14.8)	15.2 (4.6)
ADII					
Quartile 1	445 (25)	87.0 (23.0)	24.9 (6.3)	45.5 (15.9)	16.7 (5.5)
Quartile 2	443 (25)	83.6 (24.9)	24.0 (6.8)	43.2 (17.3)	16.3 (5.3)
Quartile 3	444 (25)	85.7 (24.1)	24.3 (5.3)	45.4 (17.2)	16.1 (5.1)
Quartile 4	445 (25)	94.9 (23.6)	25.5 (6.2)	51.6 (16.7)	17.8 (5.6)
HEI-2015					
Quartile 1	445 (25)	85.3 (24.5)	24.5 (6.7)	44.3 (16.7)	16.4 (5.6)
Quartile 2	444 (25)	84.8 (22.7)	24.1 (6.0)	44.5 (16.2)	16.3 (5.1)
Quartile 3	444 (25)	88.5 (24.9)	24.6 (6.4)	47.0 (17.6)	16.9 (5.5)
Quartile 4	444 (25)	92.6 (24.2)	25.5 (6.4)	49.8 (17.2)	17.3 (5.4)

AA indicates associate of arts.

The median (interquartile range) of blood metals were 0.38 μg/L (0.35), 14.70 μg/L (11.70), and 8.74 μg/L (4.06) for cadmium, lead, and manganese, respectively. Cd concentrations were higher in women and older participants (≥ 80 years) and in participants who were in the 1st quartile groups of the HEI-2015 and the ADII scores (Table 2). Pb concentrations were higher in men and older participants (≥ 80 years) and in participants who were in the 1st quartile groups of the HEI-2015 and the ADII scores (Table 2). In contrast to other metals, blood Mn concentrations were higher in participants in the 4th quartile groups of the HEI-2015 and the ADII scores.

**Table 2.** Median (interquartile range) levels of blood metals according to characteristics of study participants, NHANES 2011–2014

		Blood cadmium (μg/L)	Blood lead (μg/L)	Blood manganese (μg/L)
Characteristics of participants	n (%)	Median (IQR)	Median (IQR)	Median (IQR)
Gender				
Male	869 (49)	0.35 (0.20–0.57)	16.60 (11.50–24.80)	8.37 (6.38–10.40)
Female	908 (51)	0.40 (0.27–0.62)	13.30 (9.30–18.90)	9.08 (7.27–11.50)
Age at screening (years)				
60–69	957 (54)	0.35 (0.24–0.58)	13.70 (9.60–20.90)	8.79 (7.06–10.99)
70–79	543 (31)	0.38 (0.26–0.59)	15.30 (10.40–21.70)	8.71 (7.06–11.18)
≥80	277 (15)	0.45 (0.29–0.65)	17.30 (11.60–25.60)	8.52 (6.39–10.87)
Education level				
Less than 9th grade	199 (11)	0.38 (0.25–0.62)	14.30 (8.80–23.90)	8.90 (7.08–10.63)
9–11th grade	232 (13)	0.44 (0.27–0.75)	15.40 (10.90–24.50)	8.40 (6.82–10.49)
High-school graduate	431 (24)	0.39 (0.24–0.66)	14.00 (10.10–23.10)	8.44 (6.82–10.78)
Some college or AA degree	498 (28)	0.38 (0.26–0.57)	14.50 (10.10–20.40)	8.61 (6.91–10.81)
College graduate or above	417 (24)	0.34 (0.25–0.51)	14.90 (10.60–21.10)	9.17 (7.38–11.51)
Poverty to income ratio				
Quartile 1	408 (23)	0.42 (0.26–0.73)	15.25 (10.10–23.90)	8.92 (7.13–11.51)
Quartile 2	410 (23)	0.38 (0.25–0.63)	14.70 (10.20–22.00)	8.75 (6.88–10.87)
Quartile 3	405 (23)	0.38 (0.25–0.58)	13.80 (10.10–21.00)	8.63 (6.99–10.49)
Quartile 4	413 (23)	0.34 (0.24–0.50)	14.90 (10.40–20.60)	8.72 (6.96–11.24)
Missing	141 (8)	0.41 (0.26–0.67)	14.20 (9.50–21.20)	8.56 (6.8–10.92)
Race/Hispanic origin				
Mexican American	137 (8)	0.32 (0.23–0.45)	11.50 (8.50–16.30)	9.05 (7.75–11.11)
Other Hispanic	181 (10)	0.36 (0.25–0.60)	13.70 (9.50–20.00)	9.33 (7.65–11.54)
Non-Hispanic White	897 (50)	0.37 (0.25–0.58)	14.70 (10.20–22.00)	8.70 (6.91–10.87)
Non-Hispanic Black	417 (24)	0.38 (0.24–0.60)	15.90 (11.20–24.00)	7.65 (6.28–9.76)
Other race—including multiracial	145 (8)	0.56 (0.37–0.82)	15.20 (10.40–20.40)	11.24 (8.87–13.11)
ADII				
Quartile 1	445 (25)	0.41 (0.26–0.68)	15.70 (10.40–24.10)	8.51 (6.84–10.63)
Quartile 2	443 (25)	0.40 (0.25–0.62)	15.20 (10.10–22.70)	8.53 (6.75–10.41)
Quartile 3	444 (25)	0.36 (0.25–0.59)	14.70 (10.40–21.50)	8.73 (6.87–11.23)
Quartile 4	445 (25)	0.36 (0.24–0.52)	13.70 (9.90–19.10)	9.24 (7.52–11.42)
HEI-2015				
Quartile 1	445 (25)	0.42 (0.25–0.68)	15.35 (10.30–23.40)	8.68 (6.91–10.55)
Quartile 2	444 (25)	0.38 (0.25–0.62)	14.75 (10.20–22.40)	8.57 (6.85–10.91)
Quartile 3	444 (25)	0.38 (0.25–0.60)	15.50 (10.70–22.20)	8.76 (6.85–10.93)
Quartile 4	444 (25)	0.35 (0.25–0.51)	13.60 (9.65–19.45)	9.03 (7.25–11.41)

AA indicates associate of arts.

### Associations between blood metals mixture and cognitive performance scores

**Figure 1.**
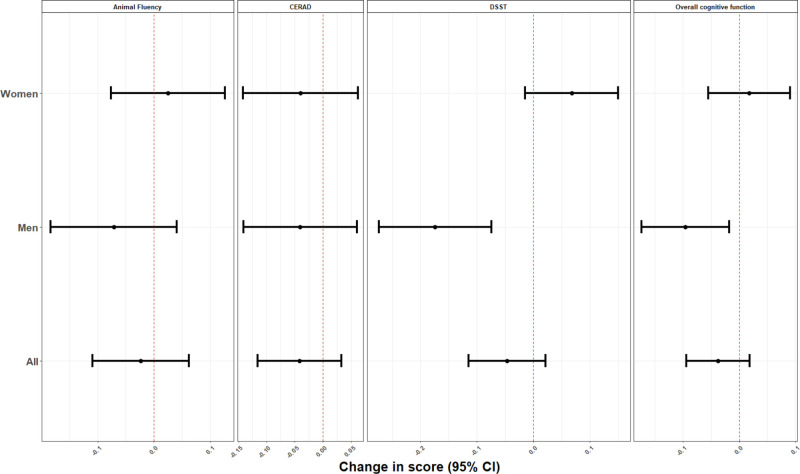
Overall and sex-specific adjusted beta coefficients (95% CI) for the overall cognitive function score and the specific cognitive performance tests, for increase in all blood metals by one quartile, NHANES 2011–2014. Model was adjusted for age (years), gender (except for stratified analysis on this variable), race/ethnicity (Mexican American, other Hispanic, Non-Hispanic White, Non-Hispanic Black, other race), language of the sample person interview instrument (English, Spanish), educational level (less than high school, high school, higher than high school), smoking status, marital status, poverty to income ratio (quartiles), and healthy eating index.

In mixture analyses using quantile G-computation, increasing blood concentrations of all metals by one quartile was associated with a decrease in overall cognitive performance (β = –0.04; 95% CI = –0.09, 0.02), CERAD (β = –0.04; 95% CI = –0.12, 0.03), and DSST (β = –0.05; 95% CI, –0.11, 0.02) test scores (Figure 1). No pattern of associations was observed for Animal Fluency test scores (β = –0.02; 95% CI = –0.10, 0.06). The individual weights for each chemical mixture component, as determined by the quantile G-computation approach, were presented in Figure S1 (http://links.lww.com/EE/A173). Lead is the only metal that had a positive weight in each of the cognitive tests and of the two metals that have negative weights, cadmium had the highest percentage except for the DSST test.

In sex-stratified analyses, we observed stronger negative associations in men and slightly positive associations in women. For instance, increasing blood concentrations of all metals by one quartile was associated with lower overall cognitive performance (β = –0.10 SD; 95% CI = –0.17, –0.02) and the DSST test scores (β = –0.17 SD; 95% CI = –0.27, –0.07) in men, whereas the associations in women were positive ([β = 0.02 SD; 95% CI = –0.05, 0.09] and [0.07 SD; 95% CI, –0.01, 0.15], *P* of heterogeneity: 0.03 and < 0.001, respectively, for overall and DSST test scores). Using traditional multivariable regressions to analyze each metal separately, Cd and Mn showed negative associations with the overall cognitive performance scores, CERAD, and DSST. Again, the associations were stronger in men for Pb and Mn with the DSST score, and Pb with the overall cognitive performance scores; *P* of heterogeneity < 0.10 for all (see Supplementary Table 1; http://links.lww.com/EE/A173).

### Dietary intake-specific effects of blood metals mixtures concentrations on cognitive performance scores

**Figure 2.**
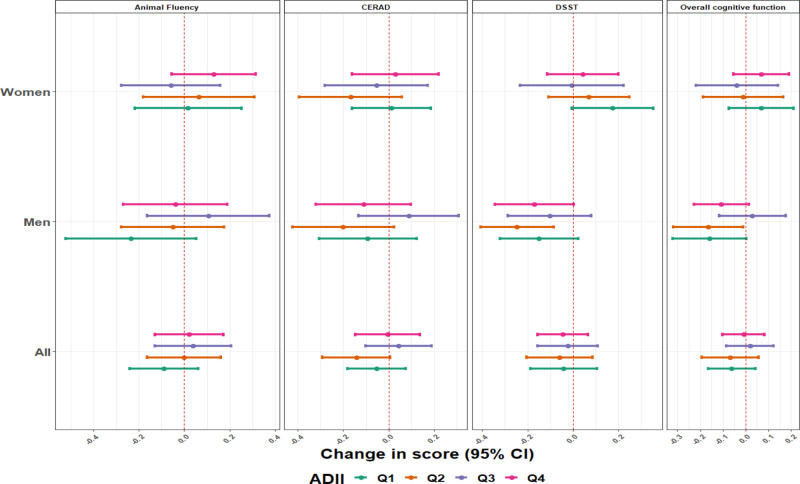
Estimates (95% CI) of the associations of a one quartile increase in the metal mixture and overall cognitive function score and specific cognitive performance tests, by sex and ADII score, NHANES 2011–2014. Model was adjusted for age (years), race/ethnicity (Mexican American, other Hispanic, Non-Hispanic White, Non-Hispanic Black, other race), language of the sample person interview instrument (English, Spanish), educational level (less than high school, high school, higher than high school), smoking status, marital status, poverty to income ratio (quartiles), and healthy eating index. All *P* values for between ADII quartile tests of heterogeneity were >0.10 except for men (*P* of heterogeneity: 0.09 and 0.07 for the overall cognitive test and the animal fluency test scores, respectively).

**Figure 3.**
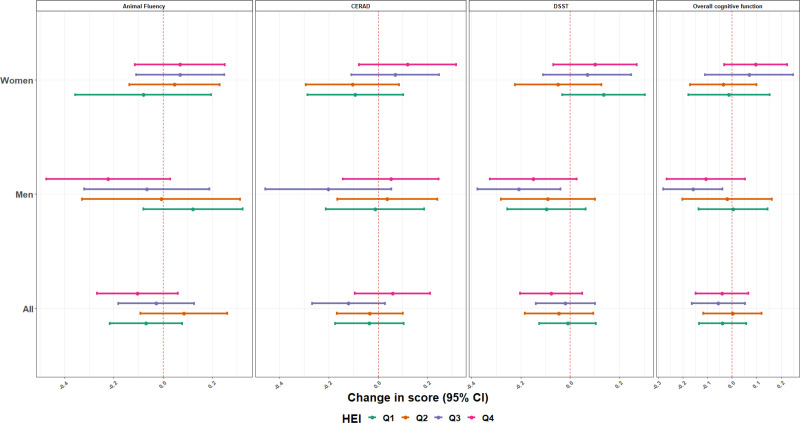
Estimates (95% CI) of the associations of a one quartile increase in the metal mixture and overall cognitive function score and specific cognitive performance tests, by Sex and HEI-2015, NHANES 2011–2014. Model was adjusted for age (years), race/ethnicity (Mexican American, other Hispanic, Non-Hispanic White, Non-Hispanic Black, other race), language of the sample person interview instrument (English, Spanish), educational level (less than high school, high school, higher than high school), smoking status, marital status, poverty to income ratio (quartiles), and ADII. All *P* values for between the HEI quartile tests of heterogeneity were >0.10 except for the overall cognitive test, the animal fluency test, and the CERAD test scores in women (all *P* of heterogeneity < 0.05) and the animal fluency test score in men (*P* of heterogeneity = 0.03).

The analyses stratified by diet quality index scores overall, and for women and men are presented in Figure 2 for the adapted dietary index and Figure 3 for the healthy eating index. The associations were more pronounced in the lower quartiles of the dietary inflammatory scores, which represent, high pro-inflammatory diet. We found null or positive though imprecise associations in participants with a high anti-inflammatory diet, especially the third quartile of the ADII score. This pattern of associations was more apparent in men. For example, blood metal mixture concentrations were inversely associated with the overall cognitive test scores among men with the pro-inflammatory diet (β = –0.16; 95% CI, –0.32, 0.00) for quartile 1, while among men with more anti-inflammatory diet, the associations were mainly null ([β = 0.03; 95% CI = –0.12, 0.18]; *P* of heterogeneity: 0.09 for quartile 3) (Figure 2). Blood metal mixture concentrations was also inversely associated with the animal fluency test scores among men with more pro-inflammatory diet (β = –0.23; 95% CI, –0.52, 0.05) for quartile 1, while among men with more anti-inflammatory diet, the associations were positive or nulls ([β = 0.11; 95% CI, –0.16, 0.37] and [β = –0.04; 95% CI = –0.27, 0.19]; *P* of heterogeneity: 0.071 and 0.066; for quartiles 3 and quartile 4, respectively) (Figure 2). We did not observe any association between blood metals mixture concentrations with the cognitive function test scores for women (Figure 2).

When the healthy eating index is considered; overall, we did not identify any pattern of association (Figure 3). However, in women, blood metal mixture concentrations was positively associated with the overall cognitive test scores among those with high-diet quality (β = 0.10; 95% CI = –0.03, 0.23) for quartile 4, while among those with low-quality diet, the associations were null or negative ([β = –0.01; 95% CI, –0.18, 0.15] and [β = –0.04; 95% CI, –0.17, 0.09]; *P* of heterogeneity: 0.04 for quartiles 1 and 2, respectively) (Figure 3). The same pattern of association was observed for the associations between blood metal mixture concentrations and the animal fluency and the CERAD tests scores. For men, we did not identify any pattern of association, except for the association between blood metal mixture concentrations and the animal fluency. An inverse association was observed in men with high-diet quality (β = –0.22; 95% CI, –0.47, 0.03) for quartile 4, and a positive association in those with the low-quality diet (β = 0.12; 95% CI, –0.08, 0.32 for quartile 1; *P* of heterogeneity: 0.03).

When the models were unweighted, we observed the same pattern of associations (Figs. S2–S4; http://links.lww.com/EE/A173). However, using an alternative definition of dietary inflammatory index showed different results. The associations between blood metals mixture and cognitive function were more pronounced in women from quartile 1 of the DII, while these associations were more pronounced in men from quartile 1 of the ADII (Fig. S5; http://links.lww.com/EE/A173). Analyses restricted to nonsmokers (46% of the total sample) showed the same pattern of associations as the analysis on the entire population.

## Discussion

In this cross-sectional study of US adults ≥60 years old, we found that concentrations of metals and metal mixtures were negatively associated with cognitive performance test scores in men but not in women. In addition, we found that an anti-inflammatory diet may mitigate the effects of metals. To our knowledge, this is the first study to investigate the potential effect of dietary modification of the association between blood metals concentrations and cognitive performance test scores in older adults.

The trace metals examined here had been previously evaluated for their effect on cognitive functions. For example, a study in an older adults population with the same NHANES data reported an inverse association between blood Cd concentrations and cognitive function scores.^4^ Two other studies in older adults in Brazil^44^ and China^45^ reported inverse associations between blood Cd concentrations and cognitive function. Manganese and lead have also been associated with diminished cognitive function in older adults. A recent meta-analysis showed that higher blood Pb concentrations are associated with poorer verbal abilities, visuospatial abilities, memory, attention, and psychomotor function.^5^ For the association between adult’s blood Mn and cognitive function, few studies reported inverse associations.^46,47^ Only a few of these studies have explored sex-specific associations and none evaluated the potential effect modification by diet in the association between metals and cognitive scores. One of the main differences of these studies compared to ours is that they focused on specific metals and did not take into account the simultaneous exposure to several metals and the potential joint associations of these metals with cognitive function.

Inflammation and oxidative stress are two key mechanisms for metal neurotoxicity. Acute high-level exposure or long-term persistent low-level exposure to Cd interferes with the antioxidant defense system and leads to oxidative stress in neuronal cells^48^ and brain endothelial cells.^49^ The mechanism by which Pb exposure affects cognition in older adults has yet to be revealed, but the existing reports suggest that the presence of Pb in the brain causes a potential pro-inflammatory effect on the central nervous system and neuronal death may be related to the production of various cytokines and chemokine.^15,50^ For Mn, the underlying mechanisms include oxidative stress and mitochondrial dysfunction, autophagy dysregulation, accumulation of intracellular toxic metabolites, and apoptosis.^51,52^ Excess manganese can impair manganese superoxide dismutase activity, thus increasing reactive oxygen species production and eventually leading to oxidative stress and mitochondrial dysfunction.^53,54^

A number of studies have provided evidence that diet plays a role in the pathogenesis of many neurological diseases such as Alzheimer’s disease, Parkinson’s disease, and multiple sclerosis.^55–57^ In our study, we observed that adherence to an anti-inflammatory diet can attenuate the potential adverse effect of higher metals concentrations on cognitive performance test scores. In older adults, no study has evaluated this potential effect modification of diet in the association between blood metals concentrations and cognitive performance test scores.

Trace elements exhibit human toxicity and disease through inflammation and oxidative stress. Thus, it is conceivable that nutrients that can contribute to cellular oxidative stress also can exacerbate or amplify environmental toxicity. On the other hand, nutrients that have antioxidant or anti-inflammatory activity could reduce or prevent compromised health or disease induction from environmental pollutants. For example, antioxidants such as vitamin C supplementation in lead-exposed animals significantly reduce blood, liver, and renal lead levels and associated biochemical changes.^58^ The main hypothesis is that vitamin C decreases the intestinal absorption of lead, possibly by reducing ferric iron to ferrous iron in the duodenum and increase the availability of iron, which competes with lead for intestinal absorption.^59^ Folic acid supplementation can lower blood arsenic concentrations,^60^ and plant-derived bioactive nutrients can lower cardiovascular and cancer risks linked to pollutant exposures.^61^ Data also indicate that bioactive food components such as polyphenols and omega-3 polyunsaturated fatty acids may scavenge the reactive oxygen species and free radicals before they can activate pathways in pathogenesis of diseases and prevent or decrease toxicant-induced inflammation.^60,62,63^

Our study has several notable strengths, including the analysis of a nationally representative sample, the use of objective laboratory values of blood metals concentrations measurement and validated tools for cognitive performance tests. In addition, the ADII we used was developed on the basis of nutritional rationale. First, the dietary inflammatory weights of dietary components are multiplied by the standardized energy-adjusted intake to reduce the between-person variation. Second, the intakes of all components are standardized to avoid the possibility that the variation in the ADII was solely driven by a few dietary components with a large range in intake. Finally, several components are excluded to avoid an overestimation of the inflammatory effects of ethanol, fat, and energy. Thus, beer, wine, liquor, and total fat were excluded. Dietary inflammatory weight for ethanol was assumed to be zero when the intake of ethanol was >40 g/d because the intake of ethanol is not likely to be anti-inflammatory when an intake is >40 g/d.^34^ Our study also had several limitations. First, NHANES is a cross-sectional study and therefore, the results cannot support causal inferences about the relationships between blood metals concentrations and cognitive performance test scores. In addition, reverse causality is possible given the cross-sectional design; however, it is improbable that such bias would explain our results. Second, as the dietary consumption data used to calculate ADII relies on participants’ ability to recall and accurately self-report dietary intake, a certain degree of under- or over-reporting can never be ruled out. However, NHANES has a long history of collecting nutrition data (since the 1960s) and continues to incorporate improvements to refine their dietary methodology. Third, as suggested in several studies, the overall health effects and especially effect on cognitive function tests scores can be seen more clearly if the measurements of metals used were indicators of cumulative chronic or long-term exposure, such as bone for lead level and kidney for cadmium level, rather than concurrent measurements in blood that only reflect recent exposure.^12,64,65^ However, we do not have data on all these metals measured in the organs where they are stored as a proxy for long-term exposure in this wave of the NHANES study. Overall, future studies should include longitudinal data, particularly on diet, and consider metals biomarkers of chronic exposures as a mixture and thus better characterize the associated risk.

## Conclusions

Our findings suggest that blood concentrations of metals mixture was negatively associated with cognitive performance test scores among older adults in the United States, especially in men. This is in line with previous findings of adverse associations between specific metals and some domains or overall cognitive test scores in predominantly male studies. This result contributes to the existing literature on the health effects of short-term exposures to metals. In addition, we found that adherence to an anti-inflammatory diet may prevent these adverse cognitive effects at least in some domains. Still, further research is needed to confirm the relationship of exposure to metals, nutrition, and cognitive performance. If confirmed, dietary patterns that ensure a sufficient intake of anti-inflammatory parameters such as vitamin C, fibers, beta carotene, and caffeine should be recommended for people at risk of exposure to toxic metals. Even if studies showed that there is a decrease in exposure to these metals as a result of various environmental health policies, a strategy based on diet could provide a potential complementary and efficient approach to counteract the potential effects of low levels of environmental pollutants.

## Conflicts of interest statement

The authors declare that they have no conflicts of interest with regard to the content of this report.

## Acknowledgments

We thank the Philippe Foundation for their contribution to and ongoing support of scientific and medical activities and research by facilitating Franco-American exchanges.

## Supplementary Material


