# Supplementary material for: Association between blood metals mixtures concentrations and cognitive performance, and effect modification by diet in older US adults
Source: Environ Epidemiol. 2022 Jan 25;6(1):e192. doi: 10.1097/EE9.0000000000000192 (PMC8835643; doi:10.1097/EE9.0000000000000192)
Supplement: Supplementary file 1 [file ee9-6-e192-s001.docx]

**Supplementary Table 1 Adjusted beta coefficients (95% CI) for the overall cognitive function and the specific cognitive performance tests, for a tenfold increase in blood metals concentrations, NHANES 2011–2014**

|  | **Overall cognitive function** |  | **CERAD test** |  | **DSST test** |  | **Animal fluency test** |
| --- | --- | --- | --- | --- | --- | --- | --- |
| **Overall** |  |  |  |  |  |  |  |
| Blood cadmium | **-0.11 (-0.25, 0.02)** |  | **-0.13 (-0.31, 0.04)** |  | **-0.21 (-0.36, -0.05)** |  | 0.00 (-0.20, 0.20) |
| Blood lead | 0.06 (-0.09, 0.20)**^*^** |  | 0.00 (-0.20, 0.20) |  | 0.00 (-0.19, 0.19)**^*^** |  | 0.17 (-0.04, 0.37) |
| Blood manganese | **-0.24 (-0.49, 0.02)** |  | -0.20 (-0.52, 0.12) |  | -0.11 (-0.45, 0.22)**^*^** |  | **-0.39 (-0.74, -0.04)** |
| **Women** |  |  |  |  |  |  |  |
| Blood cadmium | -0.10 (-0.28, 0.08) |  | -0.17 (-0.40, 0.07) |  | -0.13 (-0.35, 0.09) |  | 0.01 (-0.24, 0.26) |
| Blood lead | 0.19 (-0.02, 0.40) |  | 0.10 (-0.19, 0.38) |  | **0.27 (0.00, 0.53)** |  | 0.20 (-0.08, 0.48) |
| Blood manganese | -0.10 (-0.42, 0.22) |  | -0.28 (-0.70, 0.14) |  | 0.30 (-0.13, 0.72) |  | -0.31 (-0.77, 0.15) |
| **Men** |  |  |  |  |  |  |  |
| Blood cadmium | -0.17 (-0.60, 0.27) |  | -0.03 (-0.57, 0.51) |  | -0.43 (-0.92, 0.06) |  | -0.04 (-0.70, 0.62) |
| Blood lead | -0.29 (-0.71, 0.13) |  | -0.26 (-0.86, 0.35) |  | **-0.70 (-1.24, -0.16)** |  | 0.08 (-0.56, 0.72) |
| Blood manganese | -0.69 (-1.50, 0.12) |  | 0.05 (-0.97, 1.07) |  | **-1.46 (-2.46, -0.45)** |  | -0.66 (-1.85, 0.54) |

**^*^** p for heterogeneity test < 0.10

Models were adjusted for age (years), gender, race/ethnicity (Mexican American, other Hispanic, Non-Hispanic White, Non-Hispanic Black, other race), language of the sample person interview instrument (English, Spanish), educational level (less than high school, high school, higher than high school), smoking status, marital status, poverty to income ratio (quartiles), and healthy eating index.


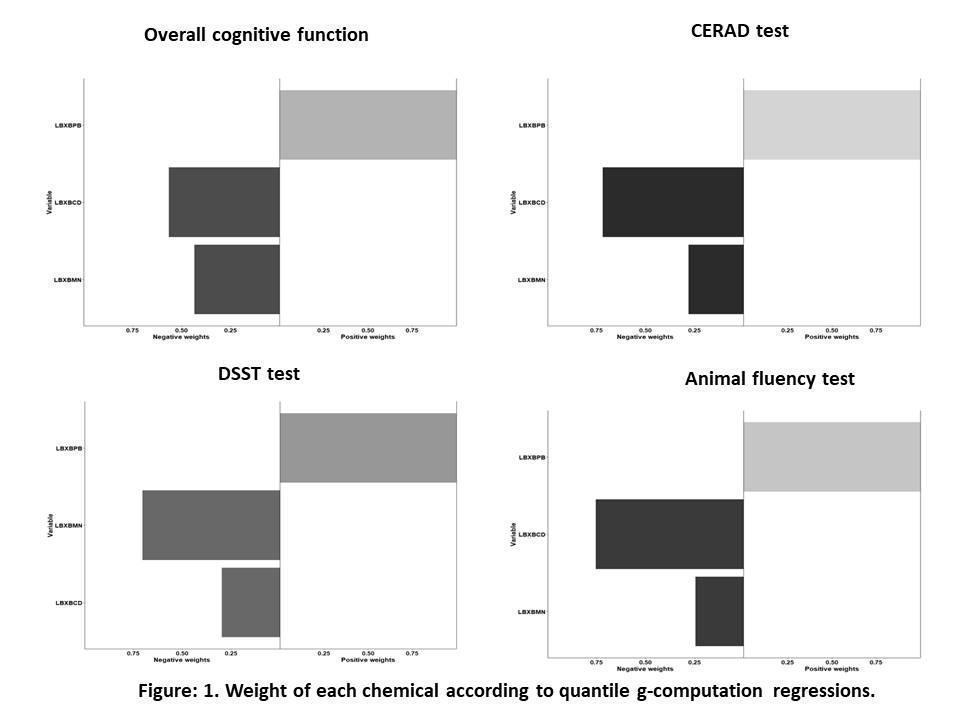


**Figure S1:** Weight of each chemical according to quantile g-computation regressions, NHANES 2011–2014.

Models were adjusted for age (years), gender, race/ethnicity (Mexican American, other Hispanic, Non-Hispanic White, Non-Hispanic Black, other race), language of the sample person interview instrument (English, Spanish), educational level (less than high school, high school, higher than high school), smoking status, marital status, poverty to income ratio (quartiles), and healthy eating index.

**
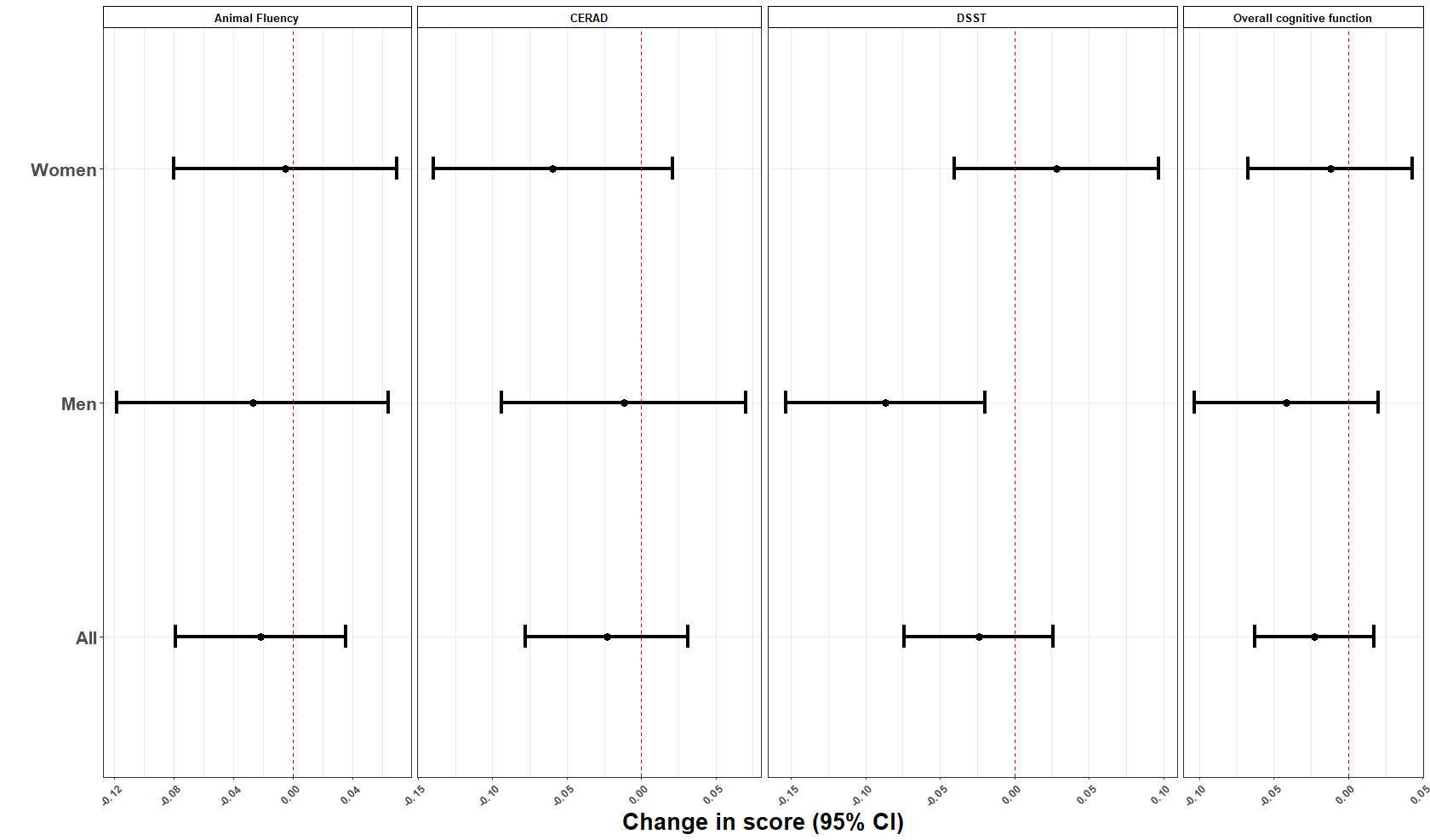
**

**Figure S2:** **Unweighted** estimates (95%CI) of the associations of a one quartile increase in the metal mixture and overall cognitive function score and specific cognitive performance tests, overall and by sex, NHANES 2011–2014.

Note: Model was adjusted for age (years), gender (except for stratified analysis on this variable), race/ethnicity (Mexican American, other Hispanic, Non-Hispanic White, Non-Hispanic Black, other race), language of the sample person interview instrument (English, Spanish), educational level (less than high school, high school, higher than high school), smoking status, marital status, poverty to income ratio (quartiles), and healthy eating index.


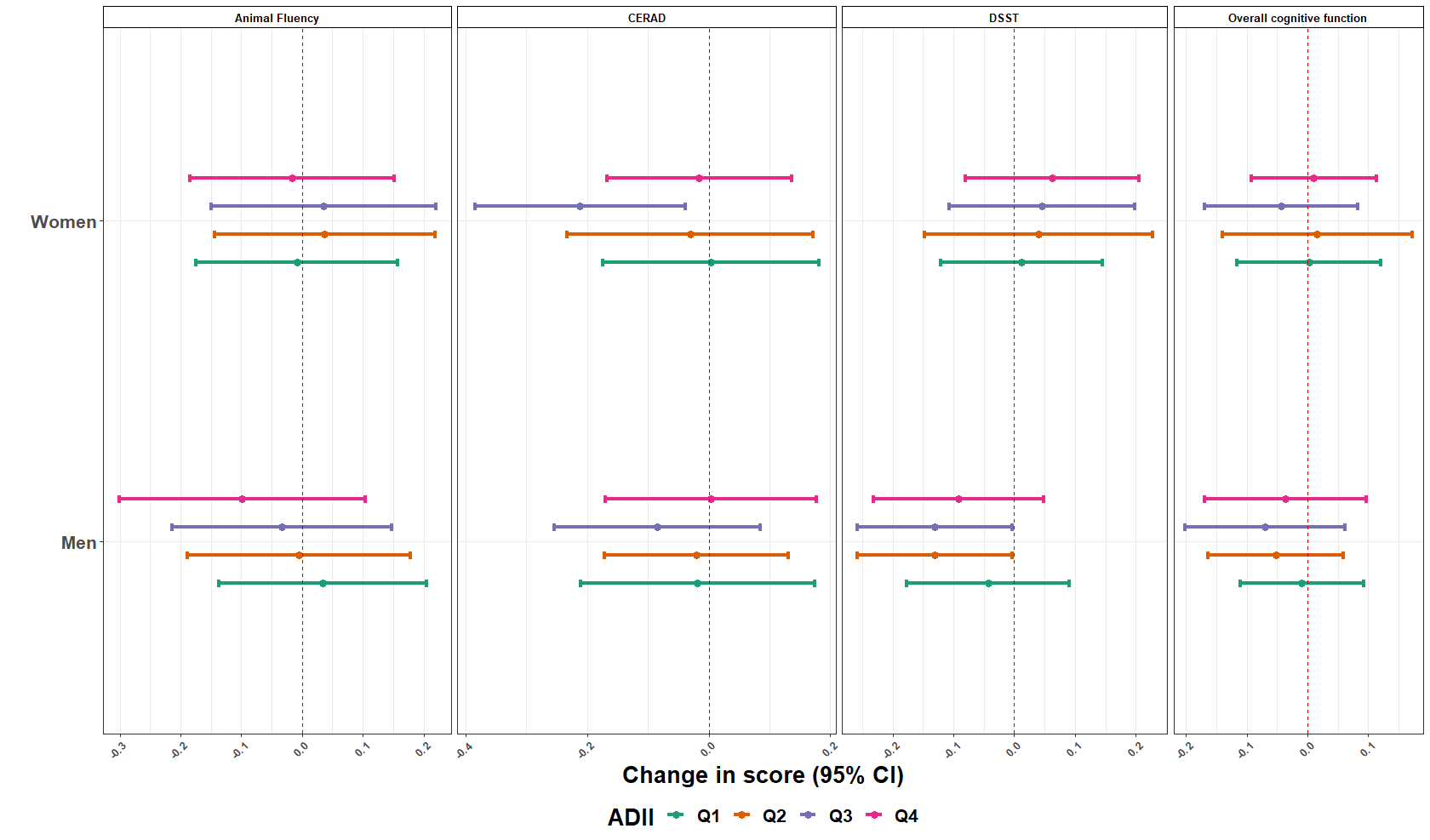


**Figure S3:** **Unweighted** estimates (95%CI) of the associations of a one quartile increase in the metal mixture and overall cognitive function score and specific cognitive performance tests, by sex and adapted dietary inflammatory index (ADII) score, NHANES 2011–2014.

Model was adjusted for age (years), race/ethnicity (Mexican American, other Hispanic, Non-Hispanic White, Non-Hispanic Black, other race), language of the sample person interview instrument (English, Spanish), educational level (less than high school, high school, higher than high school), smoking status, marital status, poverty to income ratio (quartiles), and healthy eating index. All p values for between the ADII quartile tests of heterogeneity were > 0.10.


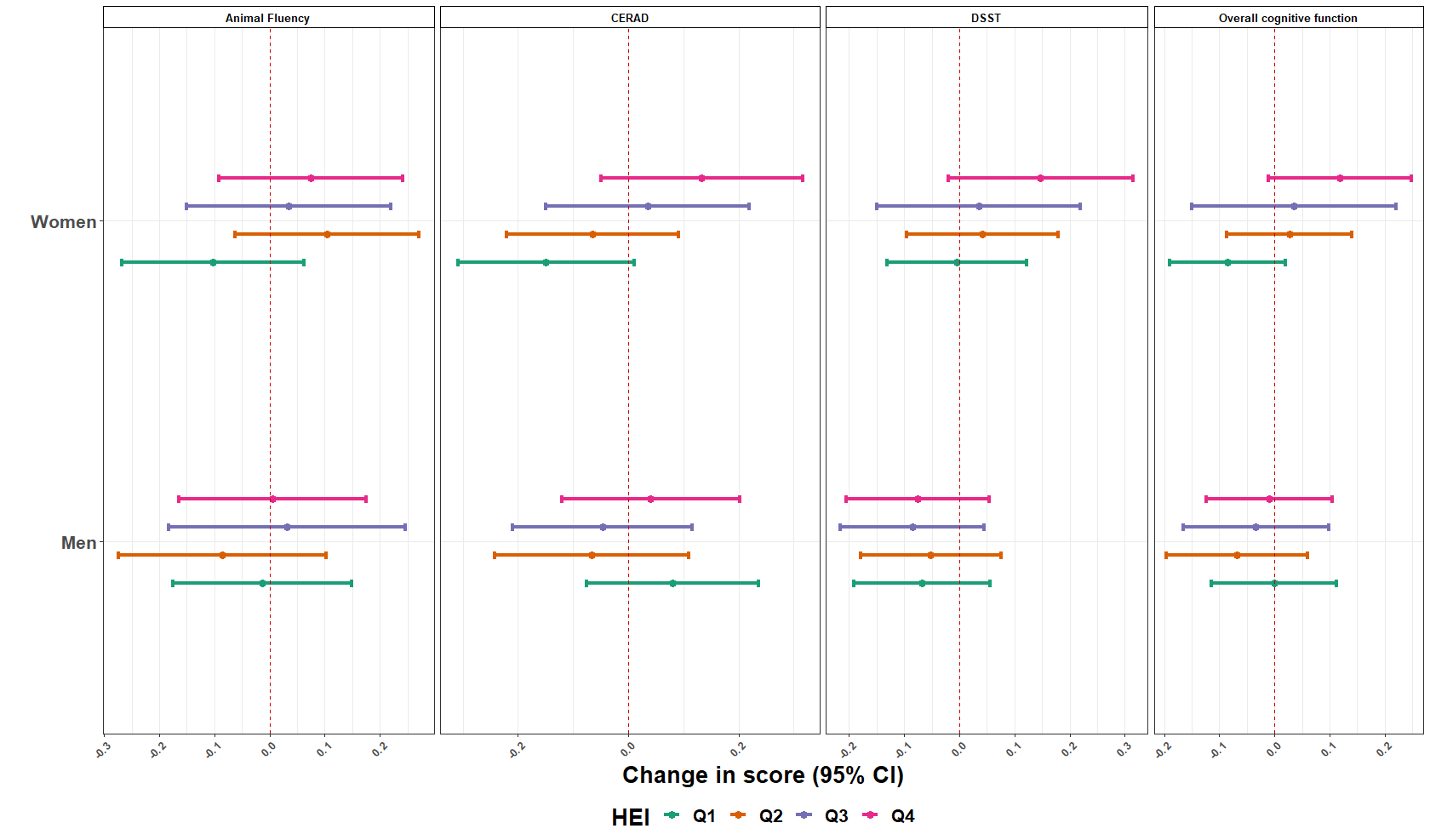


**Figure S4:** **Unweighted** estimates (95%CI) of the associations of a one quartile increase in the metal mixture and overall cognitive function score and specific cognitive performance tests, by sex and healthy eating index 2015 (HEI) score, NHANES 2011–2014.

Model was adjusted for age (years), race/ethnicity (Mexican American, other Hispanic, Non-Hispanic White, Non-Hispanic Black, other race), language of the sample person interview instrument (English, Spanish), educational level (less than high school, high school, higher than high school), smoking status, marital status, poverty to income ratio (quartiles), and adapted dietary inflammatory index. All p values for between the HEI quartile tests of heterogeneity were > 0.10 in men and < 0.10 in women.


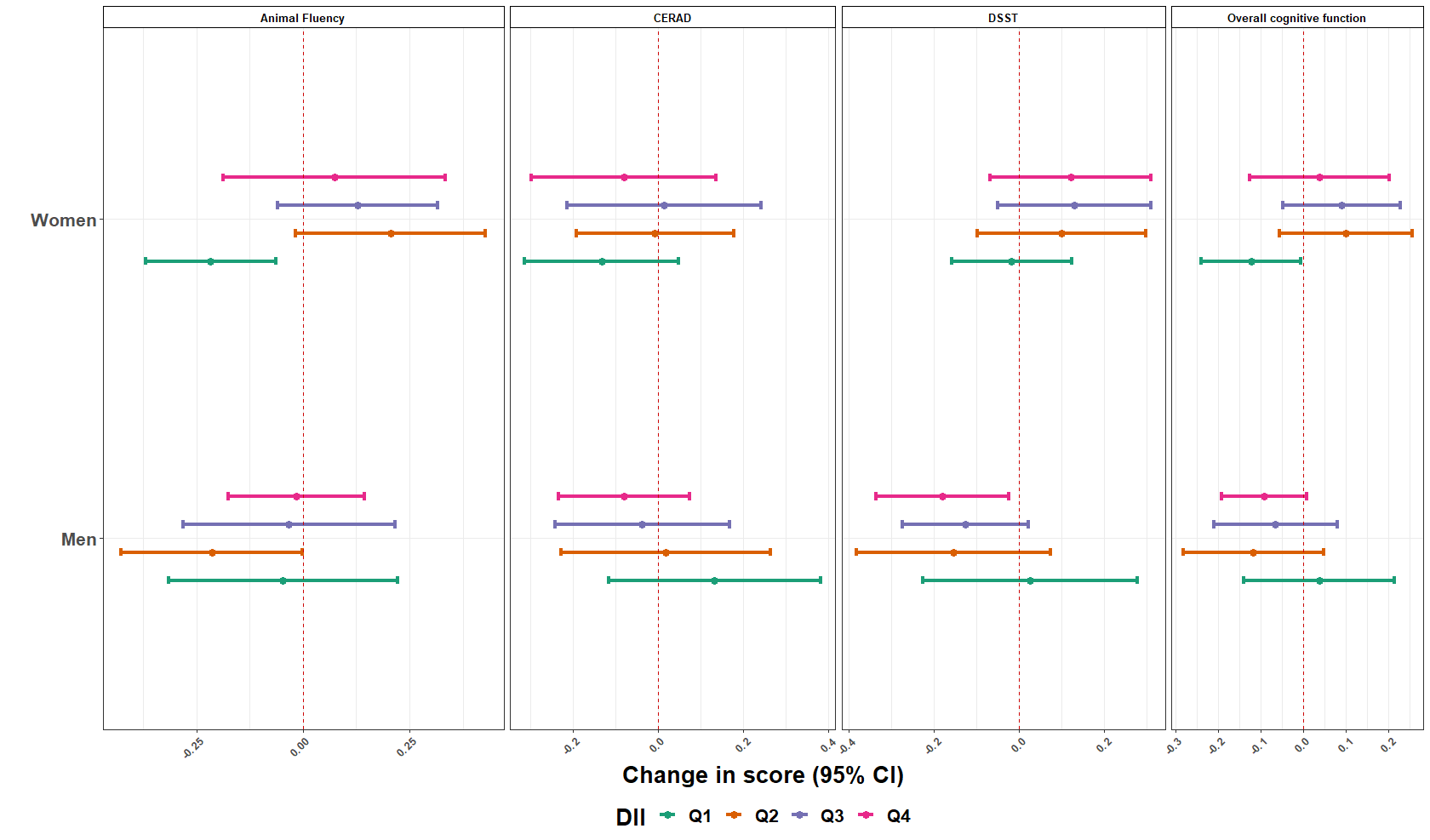


**Figure S5:** Estimates (95%CI) of the associations of a one quartile increase in the metal mixture and overall cognitive function score and specific cognitive performance tests, by sex and dietary inflammatory index (**DII) score**, NHANES 2011–2014.

Model was adjusted for age (years), race/ethnicity (Mexican American, other Hispanic, Non-Hispanic White, Non-Hispanic Black, other race), language of the sample person interview instrument (English, Spanish), educational level (less than high school, high school, higher than high school), smoking status, marital status, poverty to income ratio (quartiles), and healthy eating index. All p values for between the DII quartile tests of heterogeneity were > 0.10, except for the animal fluency test and the overall cognitive test scores (p of heterogeneity < 0.10).
